# Preliminary Insights into Geographic Variation in Venom Profiles and Functional Activities of Nigerian Snakes, *Bitis arietans* and *Naja nigricollis*

**DOI:** 10.3390/toxins18050221

**Published:** 2026-05-07

**Authors:** Akindele Oluwatosin Adeyi, Oluwatimilehin Stephen Emmanuel, Samuel Itang Itang, Babafemi Siji Ajisebiola, Mihir Kumar, Gotravalli V. Rudresha, Prasad Gopalkrishna Gond, Thomas Crasset, Damien Redureau, Fernanda Gobbi Amorim, Kartik Sunagar, Loïc Quinton

**Affiliations:** 1 Animal Physiology Unit, Department of Zoology, University of Ibadan, Ibadan P.O. Box 22133, Nigeria; eoluwatimilehin@gmail.com; 2 Evolutionary Venomics Lab, Centre for Ecological Sciences, Indian Institute of Science, Bengaluru 560012, Indiaku.mihir1998lmp@gmail.com (M.K.); rudreshabc1990@gmail.com (G.V.R.);; 3 Department of Zoology, Osun State University, Osogbo P.M.B. 4494, Nigeria; 4 Laboratory of Mass Spectrometry, MolSys Research Unit, Liège Université, 4000 Liege, Belgiumfernandagamorim@gmail.com (F.G.A.);

**Keywords:** venom variation, *Bitis arietans*, Naja nigricollis, snakebite envenoming, geographical venom phenotypes

## Abstract

Snakebite envenoming is a major yet neglected tropical disease in sub-Saharan Africa, where antivenom efficacy is critically limited by intraspecific venom variation shaped by local ecological pressures. Nigeria’s sharply contrasting Sudan Savanna (North) and Lowland Rainforest (South) provide an ideal natural system to investigate this variation, yet a comparative analysis of its medically important snakes has been lacking. We conducted an integrated proteomic and functional characterization of venoms from the puff adder (*Bitis arietans*) and black-necked spitting cobra (*Naja nigricollis*) collected in Kaduna (North) and Ibadan (South). Using high-resolution LC-MS/MS, SDS-PAGE, and biochemical assays (phospholipase A_2_, protease, fibrinogenolytic, hemolytic, and coagulation activities), we mapped region-specific venom compositions and characterized their functional activities. *Bitis arietans* displayed region-associated divergence: southern venom was enriched in serine proteases, whereas northern venom was dominated by lectins and distinct snake venom metalloproteinase isoforms. *Naja nigricollis* showed a conserved phospholipase A_2_/three-finger toxins backbone, yet southern venoms exhibited elevated snake venom metalloproteinase III and L-amino acid oxidase. These molecular differences manifested functionally, with southern *B. arietans* venom showing higher protease activity than northern *B. arietans*, whereas southern and northern *N. nigricollis* venom exhibited similar protease activity but enhanced phospholipase A_2_ activity in southern *N. nigricollis*. This work provides the first integrated proteomic and functional comparison of venoms from northern and southern Nigerian venom sample of *B. arietans* and *N. nigricollis*. While based on a limited number of individuals, the observed differences should be considered preliminary and indicative of potential regional trends rather than population-level characteristics.

## 1. Introduction

Snakebite envenoming (SBE) remains a critical public health crisis in sub-Saharan Africa, disproportionately affecting rural, agricultural communities [1]. Two medically important families dominate the pathological landscape of SBE in Nigeria: the Viperidae, notably the puff adder (*Bitis arietans*), responsible for severe local tissue necrosis, coagulopathy, and systemic hemorrhage [2,3], contributing to the often irreversible tissue damage that antivenoms frequently fail to mitigate, and the Elapidae, specifically the black-necked spitting cobra (*Naja nigricollis*), which induces potent cytotoxicity, dermonecrosis, and, in severe cases, systemic neurotoxicity [4,5]. The clinical management of envenoming hinges on the timely administration of effective antivenom. However, a pervasive challenge undermining antivenom efficacy across Africa is the profound intraspecific variation in venom composition, a phenomenon driven by a complex interplay of evolutionary pressures, including diet, predator–prey dynamics, and environmental factors [6,7,8].

Geographical divergence is a primary driver of venom variability [9,10,11,12,13]. Populations of the same species inhabiting distinct ecozones with differing faunal assemblages, climates, and altitudes can exhibit significant differences in the abundance and isoform diversity of their toxins [14,15]. These variations have direct and devastating clinical consequences. Antivenoms raised against venom from one geographical population may demonstrate markedly reduced neutralization capacity against conspecific venom from another region, which severely compromises treatment outcomes [16,17,18,19]. Understanding the scope and scale of this geographical variation in venom composition is therefore a fundamental prerequisite for developing effective, pan-regional snakebite therapies [17].

Nigeria offers a compelling model system for investigating this phenomenon. Among the major ecological zones in Nigeria are two sharply different ecozones, viz, the Guinea Savanna in the North and the humid Derived Savannah in the South [20]. These regions are characterized by distinct ecological communities that differ in vertebrate assemblages: savanna habitats in the North are dominated by small mammals and reptiles, while the southern rainforest ecosystems support a broader diversity of amphibians and mammals [21,22]. Given that venom is a trophic adaptation, it is hypothesized that the venoms of widely distributed species, such as *B. arietans* and *N. nigricollis*, would have evolved distinct compositional and functional profiles to maximize fitness in these contrasting environments. Preliminary clinical reports and anecdotal evidence suggest differences in envenoming severity between northern and southern Nigeria; yet a comprehensive, direct comparison of venoms from these venom pools using advanced omics technologies and functional analyses is conspicuously absent from the literature [7,23,24].

This study addresses this critical knowledge gap by undertaking a systematic, comparative analysis of the venom proteomes and functional activities of *B. arietans* and *N. nigricollis* from geographically disparate regions in Kaduna (North) and Ibadan (South), Nigeria. We move beyond traditional methods by integrating cutting-edge shotgun proteomics with detailed functional assays to directly link venom composition to pathological activity.

We hypothesize that the contrasting ecological pressures of the Guinea Savanna in the North and the humid Derived Savannah in the South have driven significant geographical variation in venom composition in both species, resulting in distinct venom phenotypes. To test this, we employ SDS-PAGE and high-resolution liquid chromatography–ion mobility tandem mass spectrometry (LC–IM–MS/MS) to map and compare the venom proteomes quantitatively, characterize key biochemical activities including phospholipase A_2_ (PLA_2_), proteolytic, fibrinogenolytic, hemolytic and coagulation effects to determine if compositional differences translate to functional divergence, and discuss our findings within the context of the ecology of the two regions and the practical implications for the treatment of snakebite in Nigeria.

Our findings provide a crucial molecular and functional atlas of geographical venom variation in two medically important snakes from Nigeria. This work not only advances our understanding of venom composition but also provides essential data for the rational design and selection of effective antivenoms, ultimately guiding public health strategies to mitigate the devastating impact of snakebite across diverse ecological landscapes of Nigeria.

## 2. Results

### 2.1. SDS-PAGE Protein Profile of Venoms of B. arietans from Northern and Southern Nigeria

**Figure 1:**
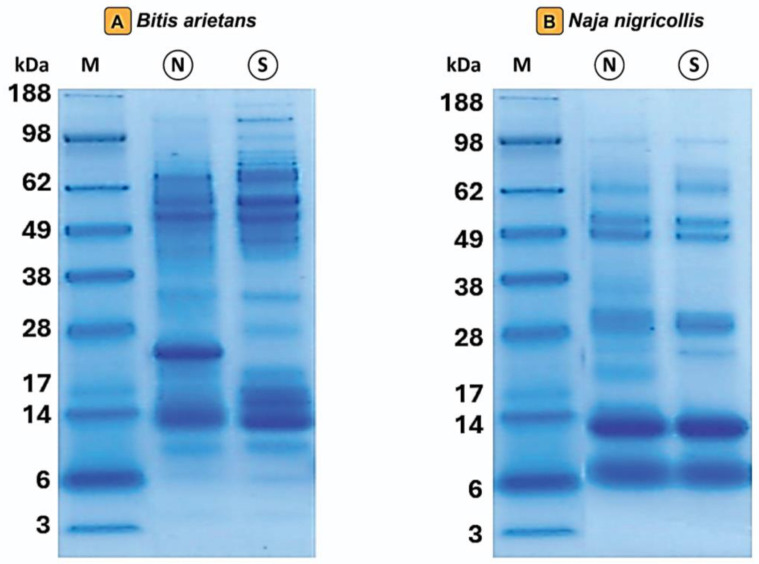
SDS-PAGE profiles of venoms from (**A**) *B. arietans* and (**B**) *N. nigricollis*. The SDS-PAGE shows the protein banding patterns of *B. arietans* and *N. nigricollis* venom, northern and southern, respectively; M: molecular-weight marker; N: northern; S: southern.

Overall, the SDS-PAGE profiles revealed clear regional differences in the venom protein banding patterns of *B. arietans* from northern and southern Nigeria (Figure 1A). The southern venom exhibited prominent low-molecular-weight (Low-MW) bands (~14–20 kDa), whereas the northern venom sample showed intense low-MW bands concentrated between ~10–14 kDa. In addition, the northern *Bitis* venom displayed a distinct band at ~22–25 kDa that was comparatively less pronounced in the southern sample. The southern venoms also showed stronger bands in the mid-MW range (~40–62 kDa) than the northern, indicating increased abundance of proteins in this mass window. Overall, the banding pattern suggested greater apparent complexity in the southern sample, reflected by broader representation across molecular-weight ranges and a higher diversity of detected components.

### 2.2. SDS-PAGE Protein Profile of Venoms of N. nigricollis from Northern and Southern Nigeria

Minor regional variations were observed in the SDS-PAGE venom protein profiles of *N. nigricollis* from the northern and southern regions (Figure 1B). Both venoms were dominated by low-MW protein bands in the ~6–9 kDa and ~10–13 kDa ranges. The similarity in these bands indicates that both venoms share a venom composition largely dominated by low-MW toxins.

In addition to these predominant components, low-intensity but distinct bands were detected in the high-MW ranges of ~27–30 kDa, ~47–50 kDa, and ~60–61 kDa in both venoms. These bands indicate the presence of additional toxin components within these molecular-weight regions. Overall, the SDS-PAGE profiles of northern and southern *N. nigricollis* venoms were broadly similar, with only minor differences in band intensity, suggesting relatively limited regional variation in the major protein components detectable by gel electrophoresis (Figure 1B).

### 2.3. Venom Protein Composition

#### 2.3.1. Protein Composition of B. arietans Venom from Northern and Southern Nigeria

**Figure 2:**
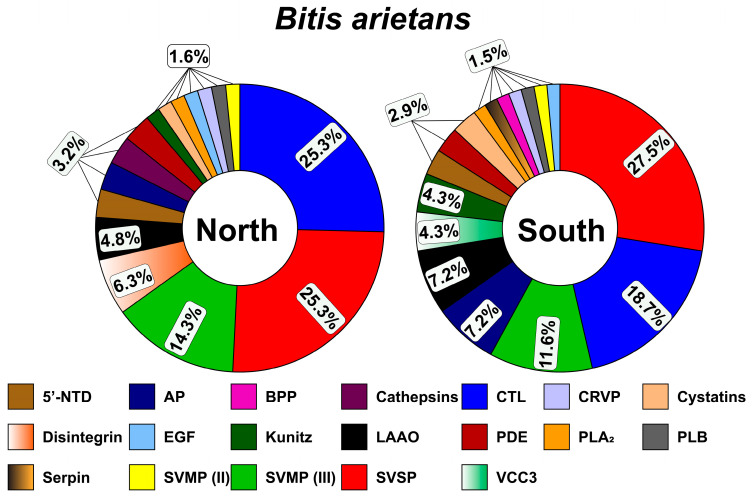
Protein composition of *B. arietans* venom from northern and southern Nigeria. Here, 5′-NTD: 5′-nucleotidase; AP: aminopeptidase; BPP: bradykinin-potentiating peptide; CTL: c-type lectin; CRVP: Cysteine-rich venom protein; EGF: endothelial growth factor; kunitz: venom kunitz-type family; LAAO: L-amino-acid oxidase; PDE: phosphodiesterase; PLA_2_: phospholipase A_2_; PLB: phospholipase B; SVMP: snake venom metalloproteinase; SVSP: snake venom serine proteinase; VCC3: venom complement C3.

Shotgun proteomic analysis of the northern Nigerian *B. arietans* venom revealed a proteome dominated by a canonical viperid toxin arsenal (Figure 2). Quantitatively, the venom was overwhelmingly characterized by a high abundance of C-type lectin [CTL; (25.3%)], snake venom serine proteinases [SVSPs; (25.3%)], and snake venom metalloproteinases III [SVMPs; (14.3%)], which collectively constituted the majority of the venom proteome. These are followed by significant contributions from disintegrin (6.3%) and L-amino acid oxidase [LAAO; (4.8%)]. Phospholipases A_2_ (PLA_2_s), along with cysteine-rich venom proteins (CRVPs), phospholipase B (PLB), and kunitz-type protease inhibitors, each contributed approximately 1.6%. Proteomic characterization of the southern Nigerian *B. arietans* venom revealed a fundamentally divergent compositional profile compared to its northern counterpart, defining a distinct venom phenotype within the same species (Figure 2). While maintaining the core viperid toxin repertoire, the southern venom exhibited an inversion of toxin family abundance, with SVSPs accounting for 27.5% of the proteome, substantially surpassing the abundance of the CTL (18.7%) and SVMPs III (11.6%). The most striking observation was the reduction in P-III SVMPs relative to the northern phenotype. This suggests a strategic modulation of venom composition, compatible with an adaptive variation in predation and defense mechanisms across ecoregions.

This compositional shift has profound clinical and ecological consequences. Ecologically, the distinct venom profiles suggest that southern *B. arietans* may have adapted to a different prey base or may rely on other defensive strategies than its northern relative.

#### 2.3.2. Protein Composition of *N. nigricollis* Venom from Northern and Southern Nigeria

**Figure 3:**
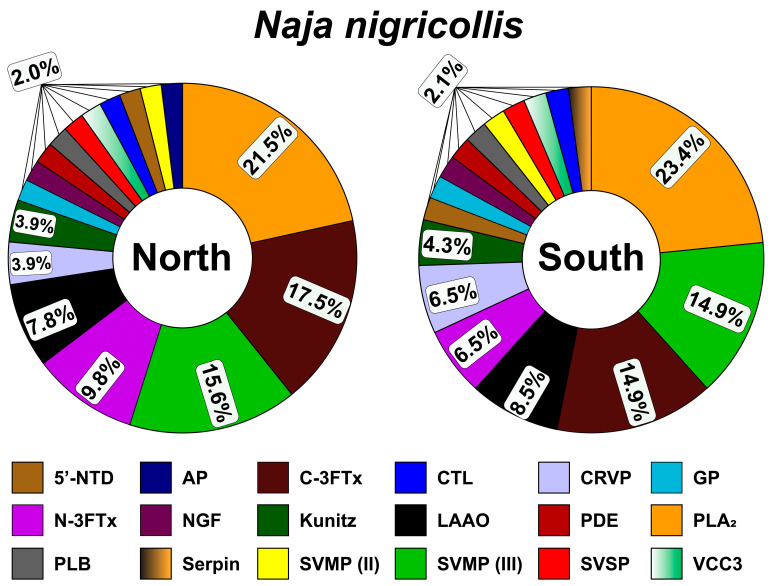
Protein composition of *N. nigricollis* venom from northern and southern Nigeria. C-3FTx and N-3FTx stand for cytotoxic and neurotoxic three-finger toxin and NGF stands for nerve growth factor. Other protein family abbreviations follow Figure 2.

Proteomic analysis of the northern Nigerian *N. nigricollis* venom revealed a highly specialized composition dominated by a high abundance of PLA_2_ (21.5%), cytotoxic three-finger toxins [C-3FTx; (17.5%)], SVMPs III (15.6%), neurotoxic three-finger toxins [N-3FTx; (9.8%)], and LAAO (7.8%; Figure 3). One significant observation was the specialization toward 3FTxs (both cytotoxins and neurotoxins), which are known to cause a wide range of pharmacological effects, primarily by targeting the nervous and cardiovascular systems. This compositional profile suggests a venom optimized for rapidly immobilizing prey against mammalian targets, where the disruption of cellular integrity and neuromuscular transmission provides a critical predatory advantage.

The proteomic profile of southern Nigerian *N. nigricollis* venom is similar to that of the northern venom (Figure 3). The southern venom exhibits dominance of PLA_2_ (23.4%), SVMPs (14.9%), C-3FTx (14.9%), LAAO (8.5%), and N-3FTx (6.5%). PLA_2_ still constitutes the majority of the venom proteome, while 3FTxs show a substantial quantitative reduction.

### 2.4. Geographic Structuring of Venom: Family-Level Differences in B. arietans and N. nigricollis

**Figure 4:**
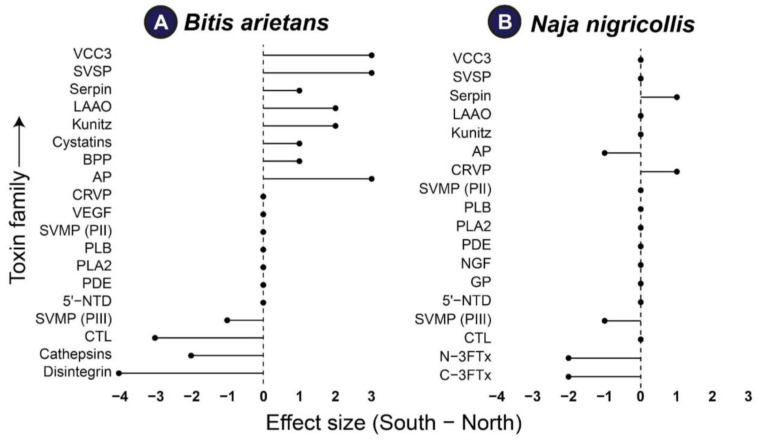
Geographic structuring of venom: family-level differences in *B. arietans* and *N. nigricollis*. In this figure, we present shifts in toxin families across the northern and southern samples. Panels (**A**) *B. arietans* and (**B**) *N. nigricollis* show the effect sizes for each toxin family. Points mark the estimated difference in abundance. The vertical dashed line at 0 denotes no difference between regions. Positive values (right of 0) show higher abundance in the southern region; negative values (left of 0) show higher abundance in the northern region. Toxin families are ordered along the y-axis. Effect sizes were calculated as the difference in abundance between the northern and southern regions. Protein family abbreviations follow Figure 2 and Figure 3.

Regional shifts in toxin family abundance (effect size = South − North) are summarized in Figure 4. Profiles for *N. nigricollis* were highly concordant between regions (Spearman ρ = 0.918, *p* = 7.6 × 10^−8^; Jaccard = 0.889, 16/18 families shared; Table S1), with most families clustering near zero. The few departures were modest and skewed toward higher northern abundance for the 3FTx families and, to a lesser extent, CTL and CRVP, whereas serpins were slightly higher in the southern sample. By contrast, *B. arietans* showed only moderate but significant concordance across regions (Spearman ρ = 0.466, *p* = 0.044; Jaccard = 0.737, 14/19 families shared; Table S1) and a wider spread of effect sizes. North-enriched families included SVMP (PIII), disintegrins, cathepsins, and CTL, while several families were South-enriched—most notably VCC3, SVSP, serpin, LAAO, kunitz, cystatins, BPP, AP, CRVP, and VEGF. Permutation tests recovered the same significance pattern, and bootstrap CIs around ρ were narrow for *N. nigricollis* and wider for *B. arietans*, indicating stronger regional coherence in the former. Overall, despite high overlap in family presence/absence, a small subset of families with pronounced regional skews appears to drive the detectable structure in each species.

#### Venom Protein Expression of *B. arietans* and *N. nigricollis* from Northern and Southern Nigeria

Volcano plot analysis identified statistically significant toxins contributing to the geographic divergence between the northern and southern venom (Figure S1). In *B. arietans*, aminopeptidase and several isoforms of P-III SVMP and SVSP were significantly upregulated in the southern venom (log_2_ FC > 2, *p* < 0.01; Figure S1A). In contrast, disintegrin and a few isoforms of P-III SVMP and SVSP were relatively enriched in the northern venom. In *N. nigricollis*, the analysis was consistent with the overall proteomic trends: SVSP, LAAO, and cobra venom factor were significantly enriched in the northern venom. Conversely, a group of PLA_2_ inhibitors, mesencephalic astrocyte-derived neurotoxic factor (MADNF), and several SVMP isoforms were among the most significantly upregulated toxins in the southern venom (Figure S1B).

### 2.5. Venom Biochemistry

Snake venoms represent intricate biochemical arsenals, composed of highly diverse toxins with distinct structural and functional profiles capable of eliciting a broad spectrum of clinical manifestations in envenomed victims. To elucidate the biochemical and pharmacological variations among these venoms, we performed a comprehensive suite of assays encompassing biochemical evaluations (PLA_2_, protease, and fibrinogenolytic activities) and pharmacological assessments (hemolytic and coagulation activities). All biochemical assays were performed using pooled venoms, with technical replicates reflecting assay reproducibility rather than biological replication.

#### 2.5.1. PLA_2_ Activity of Venoms of *B. arietans* and *N. nigricollis* from Northern and Southern Nigeria

**Figure 5:**
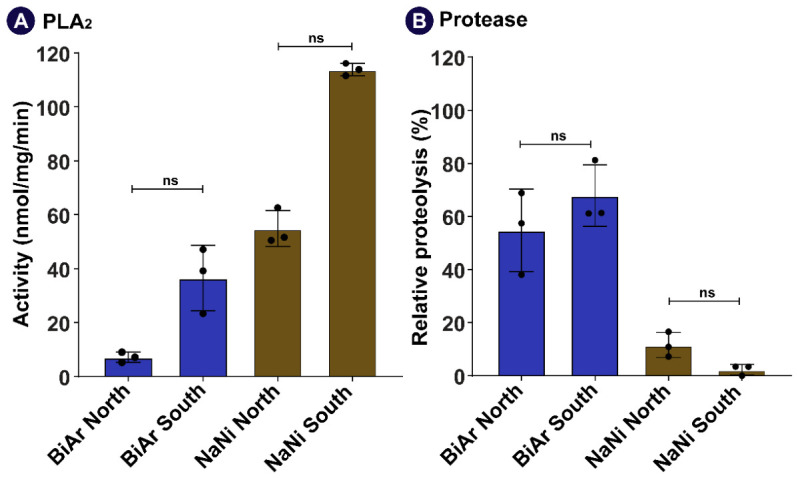
Enzymatic activities of venoms of southern and northern venom samples of *B. arietans* and *N. nigricollis*. (**A**) Phospholipase A_2_ activity and (**B**) protease activity are depicted here. All assays were performed in triplicate (n = 3; technical replicates), and the error bars represent standard deviation. Here, BiAr: *B. arietans*, NaNi: *N. nigricollis*, ns: not significant (Kruskal–Wallis test, alpha = 0.05; adjusted *p*-value = 0.61).

The PLA_2_ activity assays revealed noticeable functional differences between the northern and southern venoms of both species (Figure 5A). Overall, venoms from the South (Ibadan) exhibited higher PLA_2_ activity (*B. arietans* = 36.55 nmol/mg/min and *N. nigricollis* = 113.85 nmol/mg/min) than those from the North [Kaduna; (*B. arietans* = 7.13 nmol/mg/min and *N. nigricollis* = 54.90 nmol/mg/min)].

In *N. nigricollis*, the southern venom showed stronger PLA_2_ activity, broadly consistent with the proteome profile, indicating a substantial representation of PLA_2_ toxins in the venom composition. In contrast, the northern *N. nigricollis* venom displayed comparatively lower enzymatic activity.

Similarly, the venom of southern *B. arietans* exhibited higher PLA_2_ activity than that of the northern region. However, proteomics analysis indicated relatively higher representation of PLA_2_ components in the northern venom, suggesting that enzymatic activity may not directly reflect relative protein abundance and could be influenced by isoform diversity, toxin interactions, or non-enzymatic PLA_2_.

These assays were conducted using pooled venom; the observed differences should be interpreted as indicative of potential regional functional trends rather than definitive population-level variation.

#### 2.5.2. Protease Activity of Venoms of *B. arietans* and *N. nigricollis* from Northern and Southern Nigeria

Proteolytic activity assays revealed functional differences between venoms from northern and southern venoms of both species (Figure 5B). For *B. arietans*, the southern venom sample exhibited higher proteolytic activity than its northern counterpart. Although proteomics results indicate only modest differences in SVMP III abundance between northern and southern venom samples, protease activity was higher in the southern *B. arietans* venom. This functional divergence suggests that total proteolytic activity reflects the combined contributions of multiple enzyme families, including both SVMPs and SVSPs, rather than SVMP abundance alone. In contrast, *N. nigricollis* proteolytic activity is less than 15 for both venom samples. Northern venom shows higher relative protease activity compared to southern venom (northern = 11.57% protease activity, southern = 2.3% protease activity, Figure 5B).

#### 2.5.3. Fibrinogenolytic Activity of *B. arietans* and *N. nigricollis* Venoms from Northern and Southern Nigeria

Fibrinogenolytic analysis demonstrated functional variation that directly corresponds to the geographical divergence in venom composition (Figure S2). The most striking observation was the potent fibrinogen degradation by northern *N. nigricollis* venom, which exhibited complete cleavage of the Aα and Bβ chains of human fibrinogen within the assay timeframe. In contrast, the southern *N. nigricollis* venom showed marked reduced fibrinogenolytic potency, with almost complete degradation of the Aα chain but no degradation of the Bβ chain.

This functional hierarchy follows the same pattern: northern *B. arietans* and *N. nigricollis* > southern *B. arietans* > southern *N. nigricollis*. This mirrors the relative abundance of SVMP. Inhibition studies confirmed that both SVMPs and SVSPs were responsible for the fibrinogenolytic activity in both *B. arietans* venom, thereby requiring the combination of EDTA (ethylenediaminetetraacetic acid; SVMP inhibitor) and PMSF (phenylmethylsulfonyl fluoride; serine protease inhibitor) to inhibit fibrinogen degradation (Figure S2).

These findings underscore that the ecoregion-associated compositional differences may translate to functionally distinct coagulopathic profiles, with direct consequences for both clinical management and antivenom efficacy across ecological zones in Nigeria.

#### 2.5.4. Hemolytic and Coagulation Activity of *B. arietans* and *N. nigricollis* Venoms from Northern and Southern Nigeria

**Figure 6:**
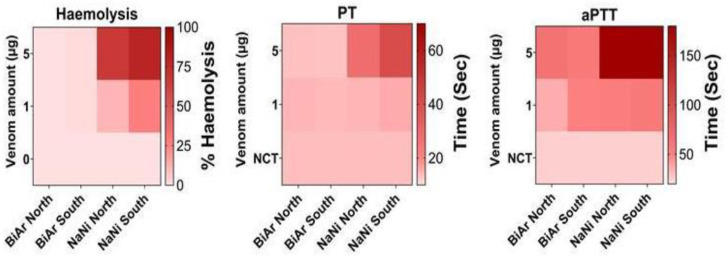
Hemolytic and coagulation activity of *B. arietans* and *N. nigricollis* venoms from northern and southern Nigeria. These heatmaps illustrate the alterations in hemolysis, PT (extrinsic pathway), and aPTT (intrinsic pathway), where the x-axis represents the snake venom tested, while the left y-axis indicates the amount of venoms, and the right y-axis indicates % hemolysis in the hemolysis heatmap and time in sec PT and aPTT. Here, NCT: normal clotting time, BiAr: *B. arietans*, NaNi: *N. nigricollis*.

Figure 6 illustrates pronounced species- and region-specific differences in hemolytic and coagulation activities among venoms of *B. arietans* and *N. nigricollis* from northern and southern Nigeria. Notably, the southern *N. nigricollis* venom induced markedly greater hemolysis than its northern counterpart, indicating enhanced membrane-disruptive potential. This pattern aligns with the elevated abundance and functional activity of PLA_2_ and C-3FTx observed in the southern venom sample (Figure 3 and Figure 5A). In contrast, *B. arietans* venoms exhibited relatively low hemolytic activity across both regions, reflecting the dominance of hemotoxic rather than direct cytolytic mechanisms in viperid envenoming. However, southern *B. arietans* venom showed a slight increase in hemolysis relative to the northern venom sample.

Coagulation assays further revealed stark functional divergence. Both northern and southern *N. nigricollis* venoms significantly prolonged clotting times in PT (prothrombin time) and aPTT (activated partial thromboplastin time) assays, confirming potent anticoagulant and consumptive coagulopathy-inducing effects characteristic of viperid venoms. The northern *N. nigricollis* venom exerted a stronger anticoagulant effect.

## 3. Discussion

Our study provides the first integrated proteomic and biochemical comparison of venoms from *B. arietans* and *N. nigricollis* sampled from the North, the arid Sudan Savanna—characterized by lower annual rainfall, higher seasonal temperature variability, and more open landscape—and South—characterized by humid lowland rainforest with higher precipitation, stable temperatures and structurally complex vegetation—of Nigeria. The results show region-associated differences in venom composition (Figure 1, Figure 2 and Figure 3) and functional activity (Figure 5, Figure 6 and Figure S2), which may be consistent with environmental, ecological, or biological influences on venom variation [17,25]. However, because these analyses were performed using pooled venoms from a limited number of individuals per region, the findings should be interpreted cautiously and regarded as preliminary rather than definitive evidence that geography alone drives venom phenotype. Instead, the present findings suggest that geographic origin may be one of several factors contributing to venom variation, alongside other variables such as diet, physiological state, and individual-level differences that contribute to the variation in venom [6,10,11,26,27,28,29,30].

The proteomic differences observed in *B. arietans* across northern and southern Nigeria (Figure 4) are consistent with previously reported intraspecific venom variation in snakes from environmentally distinct regions [11,17,25,31]. Venoms from the southern region showed relatively greater representation of proteolytic toxin families, whereas northern venoms showed comparatively higher abundance of CTL and disintegrin-associated components (Figure 2 and Figure 4; Table S2). These differences may reflect variation in ecological conditions or prey use between zones, although this remains speculative in the absence of direct dietary or environmental data. Similar associations between habitat variation and venom divergence have been reported in other viperids, including *Bothrops jararaca* in Brazil [32] and *Daboia russelii* in India [33]. In this context, our findings may be compatible with the broader concept that some venom components remain relatively conserved, while others vary more readily across regions under localized selective pressures.

A comparable pattern was also observed in *N. nigricollis*. In both regions, the venom proteome was dominated by PLA_2_ and 3FTx, suggesting a conserved elapid venom framework. At the same time, modest regional differences were detected in less abundant toxin families, including SVMP III and LAAO (Figure 3 and Figure 4). These differences may represent more variable components of the venom system, but the present dataset does not allow firm conclusions regarding the microclimatic factor responsible for these shifts. Thus, rather than demonstrating regionally fixed venom phenotypes, our results suggest that *N. nigricollis* and *B. arietans* may exhibit measurable intraspecific venom variation across Nigeria’s regions.

The possible biological relevance of this variation should also be interpreted with caution. In *B. arientans*, the relatively greater abundance of protease-associated components in southern venoms may be consistent with increased proteolytic and hemorrhagic potential, while the comparatively higher abundance of lectin-related components in northern venoms may be associated with altered hemostatic effects (Figure 5 and Figure 6). However, these proposed pathophysiological distinctions remain inferential and require validation using larger sample sets, individual-level venom analyses, and broader experimental assessment. Accordingly, the present findings should not be taken as evidence for discrete region-specific clinical syndromes, but rather as an indication that clinically relevant venom variability may exist within this species.

Similarly, the regional differences observed in *N. nigricollis* may have functional relevance, but the extent of this variation should not be overstated. The relative enrichment of SVMP III and LAAO families in southern venoms may suggest differences in tissue-damaging or oxidative properties, whereas the persistence of PLA_2_ and 3FTx dominant profiles in both regions indicate substantial conservation of the core venom architecture. Although comparable venom plasticity has been described in other elapids, including *N. mossambica* and *N. naja* [12,31,34], our results are not sufficient to conclude that southern venom possesses a definitively more damaging or clinically severe venom phenotype. Rather, they raise testable hypotheses about possible functional divergence that should be examined in future work.

These observations may also have implications for antivenom research, but such implications should be framed conservatively. Because a large proportion of major toxin families were shared across northern and southern samples, the data suggests broad similarity in the medically relevant venom framework of both species. At the same time, the regional differences observed in specific toxin families and functional activities indicate that antivenom performance could potentially vary depending on the venom sample tested. This possibility is consistent with previous studies showing that intraspecific venom variation can influence antivenom recognition and neutralization in other African and Asian snakes [7,35,36,37,38]. However, the present study did not directly test antivenom cross-reactivity or neutralization across regional venoms, and therefore any implications for antivenom efficacy should be regarded as provisional. At this stage, our findings support the need for broader geographically representative venom sampling and formal antivenom evaluation rather than immediate conclusions about the need for region-specific reformulation.

The volcano plot analyses further support the presence of measurable intraspecific proteomic differences between regions (Figure S1). These regional shifts may be consistent with ecological or region-level influences on venom composition, as reported in other snake systems [11,39,40]. Nevertheless, because the study design does not include dietary, environmental, genetic, or individual-level sampling data, it is not possible to directly attribute these differences to selective pressures associated with prey diversity or habitat alone. Thus, the present proteomic comparisons should be interpreted as evidence of regional variation rather than proof of its ecological cause.

The biochemical assays were generally concordant with the proteomic observations. In southern *B. arietans*, venoms exhibited higher total protease activity, which is consistent with their relative enrichment in protease-related toxin families like SVMPs (Figure 1 and Figure 5). This led to increased extracellular matrix degradation and local hemorrhagic damage which corresponds to previous observations in West African *Bitis* [41]. Northern venom showed comparatively lower proteolytic activity but stronger anticoagulant effects (Figure 6), which may reflect the higher abundance of lectin-associated components and their known roles in modulating hemostatic pathways [42]. These associations support the internal consistency of the dataset, although they should not be interpreted as direct evidence of distinct clinical outcomes without additional validation.

In *N. nigricollis*, southern venom showed greater PLA_2_-associated activity (Figure 3), which may be compatible with increased cytotoxin potential, whereas the northern venom appeared to favor other functional effects. However, caution is warranted in linking these experimental differences directly to geographic variation in human clinical presentation. Although prior reports have described variability in envenoming manifestations across regions [43], the present study did not assess clinical samples or patient outcomes. Therefore, the functional differences observed here are best viewed as experimentally measurable differences with possible, but as yet unconfirmed, pathophysiological significance.

Differences in fibrinogen degradation were also observed between regions (Figure S2). Both northern and southern *B. arietans* venom degraded fibrinogen chains, indicating strong fibrinogenolytic activity, although the extent and pattern of degradation varied between samples. These differences may reflect variation in the relative abundance or activity of SVMPs and SVSPs, as described in other hemotoxic snake venoms [32,44]. Likewise, *N. nigricollis* venoms also showed fibrinogenolytic activity, with some regional variation in the rate of degradation. Taken together, these findings suggest that hemostatic effects may vary between venom, but the limited sample size precludes broader conclusions about region-wide functional phenotypes.

The hemolytic and coagulation assays provide additional support for region-associated functional variation (Figure 6), particularly in *N. nigricollis*. Southern venom showed greater hemolytic activity, whereas northern venom showed stronger anticoagulant effects in our experimental system. These results may indicate differences in the balance of toxin activities between regions. However, any inference regarding increased risk of renal injury, circulatory collapse, or other systemic outcomes would be premature without in vivo or clinical corroboration. Similarly, although these findings raise the possibility that preclinical antivenom testing should include functionally diverse venoms, the present study does not directly demonstrate reduced antivenom efficacy against either regional population.

A limitation of the present study is the few numbers of individuals sampled per species per region. This reflects logistical and ethical constraints inherent to venomous snake collection. Consequently, the venom differences reported here should be interpreted as preliminary, hypothesis-generating observations rather than definitive representations of population-level venom phenotypes. Future studies incorporating larger sample sizes across multiple localities, individual-level venom profiling, and direct antivenom evaluation will be important for determining the biological and translational significance of the variation suggested by the present dataset.

## 4. Conclusions

The functional divergences observed in this study may have translational relevance, but these implications should be interpreted cautiously. The regional variation detected in toxin composition and activity suggest that envenoming outcome could differ between regions, with southern venom potentially showing greater proteolytic, hemorrhagic, and tissue-damaging effects, while northern venoms may differ in their hemostatic impact. However, these possibilities remain inferential and were not directly evaluated in clinical or in vivo experiments. Likewise, the intraspecific variation observed here raises the possibility that antivenom performance may vary depending on the venom sample used for production and testing. Because antivenoms currently used in Nigeria are produced from a limited immunogen base, further work is needed to determine whether such variation affects cross-neutralization efficacy. Accordingly, our findings support the importance of broader geographically representative venom sampling and formal antivenom evaluation across populations. These results may ultimately help inform future improvements in antivenom design and clinical management, but such applications will require validation through larger, individual-level, and preclinical studies.

## 5. Materials and Methods

### 5.1. Collection of Snake Species and Venom Extraction

Adult male wild-caught *B. arietans* and *N. nigricollis* (n = 2) same-sized individuals were captured in Kaduna, Northern region, with area coordinates of latitude 11.085541, longitude 7.719945, and Ibadan, Southern region of Nigeria, with coordinates of latitude 7.376736, longitude 3.939786 (Figure S3) with the prior permission of the Forest Research Institute of Nigeria, Federal Ministry of Environment (FR/ADMIN.328/IV/817). All protocols for snake handling were observed and carried out in accordance with the University of Ibadan Animal Care and Use Ethics Committee and in compliance with the revised ARRIVE guidelines 2.0. Venom was collected from freshly captured adult snakes into sterile containers using the method previously described [45]. The venom samples were lyophilized and stored at −20 °C prior to use. Whole venoms obtained from individuals of the same species and regions were pooled for proteomic and biochemical analyses.

### 5.2. Sample Preparation for Proteomics

Proteomic analysis of venom samples was done in the Laboratory of Mass Spectrometry (MS-Lab), MolSys Research Unit, Liege University, Belgium. Approximately 10 µg of each pooled lyophilized venom was dissolved in 13.5 µL of 50 mM NH_4_HCO_3_, pH 7.8. The samples were reduced with 1.35 µL of 110 mM dithiothreitol (DTT) for 40 min at 56 °C under shaking at 600 rpm. Then, reduced samples were alkylated for 30 min at room temperature in the dark with 1.3 µL of 250 mM iodoacetamide. After that, 1.8 µL of 110 mM dithiothreitol (DTT) was added for 10 min at room temperature in the dark to quench the reaction. The samples were then digested enzymatically using trypsin in two consecutive steps. The first step involved a 1:50 (trypsin:protein) ratio and was incubated overnight at 37 °C with shaking at 600 rpm. The second step was performed with a 1:100 ratio and incubation at 37 °C for 3 h (shaking 600 rpm). Stop reactions were conducted by acidifying the medium with 10% TFA (final concentration). Finally, the digested samples were dried under a speed vacuum. Before mass spectrometry analysis, the samples were suspended in 20 µL of 0.1% TFA for desalting on ZipTip pipette tips with C18 resin. The elution was performed by 20 µL of 0.1% TFA/ACN (50/50).

### 5.3. Shotgun Proteomics

Peptide solutions were injected on an Acquity UPLC M-Class liquid chromatography system (Waters, Milford, MA, USA) coupled with a tims TOF SCP instrument (Bruker Daltonics, Bremen, Germany) using data-independent acquisition (DIA). The LC instrument was in Trap Elute configuration with a 10 µL loop, a Symmetry C18 trap column (Waters, Milford, MA, USA; ACQUITY UPLC M-Class Symmetry C18 Trap Column, pore size: 100 Å, particle size: 5 µm, ID: 180 µm, length: 20 mm, Cat. No.: 186007496) followed by an analytic C18 column AuroraGen3 Elite (IonOpticks; Oxfordshire, UK; pore size: 120 Å, particle size: 1.7 µm, ID: 75 µm, length: 15 cm, Cat. No.: 186007496) equipped with a CaptiveSpray emitter tip. The LC method involved a 3 min trapping step at 2% ACN followed by an analytical gradient ramping from 2% to 40% ACN over 29 min. M-Class system was connected to atims TOF SCP instrument (Bruker Daltonics, Bremen, Germany) using a CaptiveSpray interface for nanoESI ionization. Ion mobility and *m*/*z* values have been calibrated using Agilent TuneMix. Ion mobility separation was performed from 1/K0 from 0.75 to 1.25 V.s/cm^2^ with a ramp of 150 ms and an accumulation time of 150 ms (duty cycle ≈ 100%). Samples were analyzed in positive mode in data-dependent acquisition (DDA) using Parallel Accumulation–Serial Fragmentation (PASEF). The number of PASEF ramps was set to 6 for a total cycle time of 1.1 s. Only precursors with a charge between 2 and 5 are selected for MS/MS analysis with a minimal intensity of 1000 cps. The target intensity was set to 15,000 cps. A precursor filter is applied in function of the *m*/*z* and the ion mobility value to target multicharged peptide precursor ions for MS/MS analysis.

### 5.4. Proteomic Data Analysis

Raw LC–IM–MS/MS data were processed using FragPipe (version 23.1; NesViLab, Alexey Nesvizhskii’s Proteome Bioinformatics Group). Protein identification for Naja venoms was performed against the “Elapidae AND Venom” database (97,831 sequences), whereas the “Viperidae AND Venom” database (35,433 sequences) was used for Bitis venoms. All databases were retrieved from UniProt, accessed on 29 September 2025. Post-translational modifications (PTMs) were specified as follows: carbamidomethylation (C) as a fixed modification, and oxidation (M), acetylation (protein N-terminus), and deamidation (N/Q) as variable modifications, with a maximum of 3, 1, and 1 occurrence(s), respectively. Enzymatic digestion was set to “strict trypsin”, allowing up to three missed cleavages. The precursor and fragment mass tolerances were both set to 20 ppm. PSM validation was performed using Percolator with the options -only-psms, -no-terminate, and -post-terminate-tdc. Protein inference was conducted using ProteinProphet with the command-line parameter -maxppmdiff 2,000,000 *.

Quantitative protein intensity values obtained from the FragPipe/IonQuant workflow were imported into Perseus (v2.1.5.0) for downstream statistical analysis. Prior to analysis, the dataset was curated by removing common contaminants, reverse sequences, and proteins identified only by site, as exported by FragPipe. Proteins were retained only if quantified at least 3 times in each group of replicates. MaxLFQ intensity values were subsequently log_2_-transformed to stabilize variance.

Missing values were handled using either imputation based on a downshifted Gaussian distribution (width = 0.3, downshift = 1.8), following standard Perseus recommendations to approximate low-abundance protein signals.

Differential Abundance Analysis (pairwise comparisons) between experimental groups (southern vs northern) was carried out using a two-sample Student’s *t*-test (250 randomizations, FDR 0.05, S0 = 0.1). The resulting *t*-test statistics and fold-changes were visualized using volcano plots generated in Perseus to highlight significantly up- and downregulated proteins.

The raw mass spectrometry proteomics data have been deposited in the Proteome Xchange consortium via the PRIDE repository under accession number PXD073787.

### 5.5. SDS-PAGE for Visualization of Venom Complexities from Different Regions

Each venom (40 µg) was mixed with 4× Laemmli buffer (Tris-HCL, pH 6.8, 130 mM; SDS 4%; Glycerol 4%; DTT, 700 mM) in a 1:3 (*v*/*v*) sample-to-buffer ratio and heated at 100 °C for 3 min. The proteins were separated using a 4–12% NuPAGE MES gel (Thermo Fisher Scientific, Waltham, MA, USA) under a constant voltage of 200 V for 45 min. A molecular marker standard containing insulin beta-chain (3 kDa), aprotinin (6 kDa), lysozyme (14 kDa), red myoglobin (17 kDa), carbonic anhydrase (28 kDa), alcohol dehydrogenase (38 kDa), glutamic dehydrogenase (49 kDa), bovine serum albumin (62 kDa), phosphorylase (98 kDa), and myosin (188 kDa) was included for reference. After the gel run, it was dehydrated using a solution of 50% ethanol and 3% phosphoric acid (*v*/*v*) for 3 h, followed by rehydration in ultrapure water (MilliQ) for 20 min. Protein bands were visualized using a gel documentation system after overnight staining with Coomassie blue (360 g/L) in an aqueous buffer containing 34% methanol (*v*/*v*), 3% phosphoric acid (*v*/*v*), and 17% ammonium sulfate (*v*/*v*). To preserve the gel, it was stored at 5 °C in a solution of 5% acetic acid (*v*/*v*).

### 5.6. Biochemical Characterization of the Snake Venoms

Protein concentration and biochemical characterizations of the venoms were conducted at the Evolutionary Venomics Laboratory, Indian Institute of Science, Bangalore, India. Protein concentration was estimated using the Bradford assay, with Bovine Serum Albumin (BSA) as the standard [46]. Biochemical and pharmacological activities were examined, and all experiments were performed in triplicate (n = 3).

#### 5.6.1. Colorimetric Phospholipase A_2_ (PLA_2_) Assay

A slightly modified version of the previously described protocol was used to evaluate the phospholipase activity of the snake venoms, utilizing the chromogenic lipid substrate 4-nitro-3-[octanoyloxy] benzoic acid (NOB; Enzo Life Sciences, New York, NY, USA) [47,48]. Briefly, 5 μg venom was incubated with 500 mM NOB substrate in a 200 μL reaction buffer (10 mM Tris–HCl, 10 mM CaCl_2_, 100 mM NaCl, pH 7.8) at 37 °C for 40 min. The reaction kinetics were monitored at 425 nm using an Epoch 2 microplate spectrophotometer (BioTek Instruments, Inc., Winooski, VT, USA). Following the same protocol, a standard curve was generated using varying concentrations of the NOB substrate (4 nmol to 130 nmol) and 4 M NaOH. From this curve, the phospholipase activity was expressed as the amount of phospholipid substrate (in nmol) cleaved per minute per mg of venom protein.

#### 5.6.2. Snake Venom Protease Assay

To evaluate the proteolytic activities of the venoms, a modified version of a previously established protocol was employed [49]. A known quantity of venom (10 μg) was incubated with azocasein substrate at 37 °C for 90 min. The reaction was terminated by adding 200 μL of trichloroacetic acid (TCA), followed by centrifugation at 1000× *g* for 5 min. An equal volume of 0.5 M NaOH was then added to the resulting supernatant, and the absorbance was measured at 440 nm using an Epoch 2 microplate spectrophotometer (BioTek Instruments, Inc., Winooski, VT, USA). Purified bovine pancreatic protease (Sigma-Aldrich, St. Louis, MO, USA) was used as a positive control to calculate the relative proteolytic activity of the venoms.

#### 5.6.3. Fibrinogenolytic Assay

Gel electrophoresis was employed to analyze the fibrinogenolytic activity of the venoms [50]. Briefly, 15 μg of human fibrinogen (Sigma-Aldrich, St. Louis, MO, USA) was incubated with 1.5 μg of venoms at 37 °C for 60 min. The reaction was terminated by adding an equal volume of sample loading buffer (1 M Tris-HCl, pH 6.8; 50% glycerol; 0.5% bromophenol blue; 10% SDS; and 20% β-mercaptoethanol) followed by heating the mixture at 70 °C for 10 min. The samples were resolved on a 15% polyacrylamide gel, and the band patterns of the fibrinogen cleavage products were compared to those of an untreated human fibrinogen control. To determine the relative contributions of snake venom serine proteases (SVSPs) and snake venom metalloproteinases (SVMPs) to the fibrinogenolytic activity, inhibition studies were conducted by pre-incubating the venom with EDTA (SVMP inhibitor) and PMSF (SVSP inhibitor). The resulting bands were visualized using the iBright CL1000 gel documentation system (Thermo Fisher Scientific, Waltham, MA, USA). Densitometric analysis was performed using the ImageJ software (version 1.54g) [51], and the results were presented using GraphPad Prism 10.

### 5.7. Hemolytic Assay

The hemolytic activity of these venoms was assessed by treating graded amounts of venoms (1 and 5 µg) with freshly isolated RBCs from human blood [33]. Briefly, RBCs were separated from plasma and resuspended in PBS (pH 7.4) after post-washing. The RBC solution and venoms were mixed in a 10:1 ratio and incubated at 37 °C for 24 h. The following day, the absorbance of the supernatant was measured at 540 nm using an Epoch 2 microplate spectrophotometer (BioTek Instruments, Inc., Winooski, VT, USA), and the relative hemolytic activities of venoms were calculated using 0.5% Triton X as a positive control (treated as 100% activity) and RBCs in buffer as the negative control. To account for any background absorbance, venom-only samples (in buffer, without RBCs) were measured and their values were subtracted from the corresponding venom + RBC readings, ensuring accurate normalization of hemolytic activity.

### 5.8. Coagulation Assays

To evaluate the effects of the venoms on the intrinsic and extrinsic coagulation pathways, activated partial thromboplastin time (aPTT) and prothrombin time (PT) were measured, respectively [52]. Freshly collected human blood samples were placed in tubes containing 2% sodium citrate (BD Vacutainer^®^, Gurgaon, Hiryana, India) and centrifuged at 3000× *g* for 10 min at 4 °C to obtain platelet-poor plasma (PPP). For PT assessment, prewarmed thromboplastin reagent (Uniplastin; Tulip Diagnostics, Mumbai, India) was mixed with 50 µL of PPP and treated with varying concentrations of the snake venoms (1 and 5 µg; n = 1). For aPTT estimation, activated cephaloplastin reagent and 0.02 M calcium chloride were mixed with 50 µL of PPP, followed by the addition of graded venom concentrations (1 and 5 µg; n = 1). The time taken for the first clot formation was recorded using a Hemostar XF 2.0 coagulometer (Tulip Diagnostics).

### 5.9. Statistics

Statistical comparisons for all biochemical assays were performed using one-way ANOVA followed by Dunn’s multiple comparisons test (compared the mean ranks of preselected pairs of columns; North vs South for both species) in GraphPad 10 (GraphPad Software, La Jolla, CA, USA, www.graphpad.com). Toxin family compositions between northern and southern venom samples of *B. arietans* and *N. nigricollis* from Nigeria were determined using abundance per family. The analysis was performed in R (R4.5.1; www.R-project.org) using the following packages: tidyverse [53], dplyr [54], readr [55], tidyr [56], forcats [57], ggplot2 [58]. Proteomic statistical analyses were performed in Perseus (v2.1.5.0) and R (v4.5.1) using explicitly defined preprocessing, normalization, and multiple-testing correction parameters.

## Data Availability

The original contributions presented in this study are included in the article/Supplementary Materials. Further inquiries can be directed to the corresponding author.

## Supplementary Materials

The following supporting information can be downloaded at: https://www.mdpi.com/article/10.3390/toxins18050221/s1, Figure S1: Differential expression of venom proteins between northern and southern venom of *Bitis arietans* and *Naja nigricollis*; Figure S2: Fibrinogenolytic activity of *B. arietans* and *N. nigricollis* venoms from northern and southern Nigerian snake samples; Figure S3: Map of Nigeria depicting various forest types and locations of snake venom collection. Table S1: Concordance between venom proteomes across northern and southern venom samples of Nigerian snakes (*B. arietans* and *N. nigricollis*); Table S2: Family-level differences in toxins between northern and southern venom samples of Nigerian snakes (*B. arietans* and *N. nigricollis*).
