# Supplementary material for: Preliminary Insights into Geographic Variation in Venom Profiles and Functional Activities of Nigerian Snakes, Bitis arietans and Naja nigricollis
Source: Toxins (Basel). 2026 May 7;18(5):221. doi: 10.3390/toxins18050221 (PMC13211584; doi:10.3390/toxins18050221)
Supplement: Supplementary file 1 [file toxins-18-00221-s001.zip › toxins-4261761_supplementary_figure.pdf]

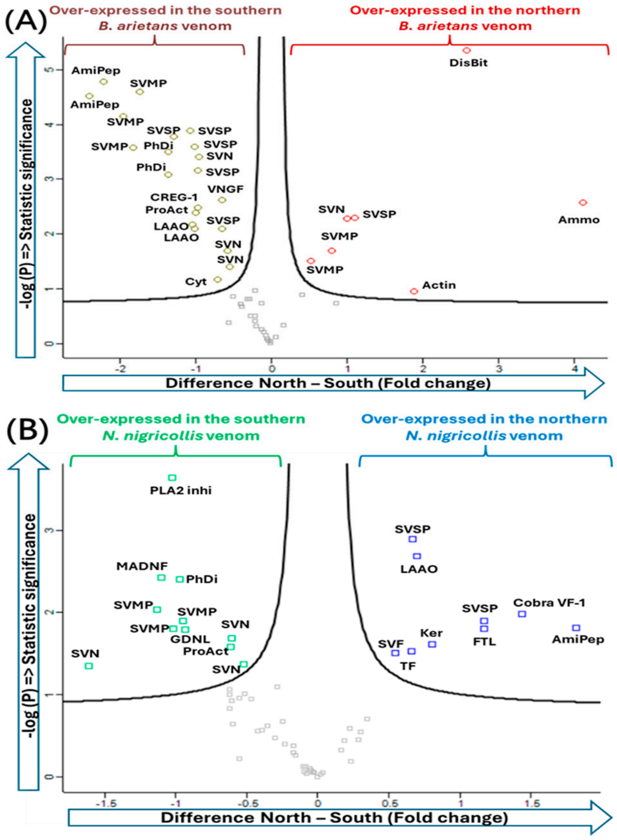

Abbreviation	Protein
Actin	Actin, cytoplasmic 2
AmiPep	Aminopeptidase
Ammo	Ammodytin L(2) variant
Cobra VF-1	Cobra venom factor 1
CREG-1	CREG1-like protein
Cyt	Cytotoxin
DisBit	Disintegrin Bitistatin
FTL	Formate--tetrahydrofolate ligase
GDNL	Glia-derived nexin-like protein
Ker	Keratin (contaminant)
LAAO	L-amino-acid oxidase
MADNF	Mesencephalic astrocyte-derived neurotrophic factor
PhDi	Phosphodiesterase
PLA2 inhi	Phospholipase A2 inhibitor PIP
ProAct	Proactivator polypeptide-like
SVMP	Snake venom metalloproteinase
SVN	Snake venom 5'-nucleotidase
SVSP	Snake venom serine protease
TF	Transferrin
VNGF	Venom nerve growth factor

Figure S1: Differential expression of venom proteins between northern and southern venom of *Bitis arietans* and *Naja nigricollis.* Volcano plots showing statistically significant differences in venom protein abundance between northern and southern Nigerian snake venom samples. (A) *Bitis arietans* and (B) *Naja nigricollis.* The x-axis represents the $\log_2$ fold change (North - South), while the y-axis indicates statistical significance ($-\log_{10}$ adjusted *p*-value). Proteins significantly enriched in the northern venoms appear on the right. Labeled points represent proteins with significant differential abundance. The table lists the protein abbreviations shown in the plots.

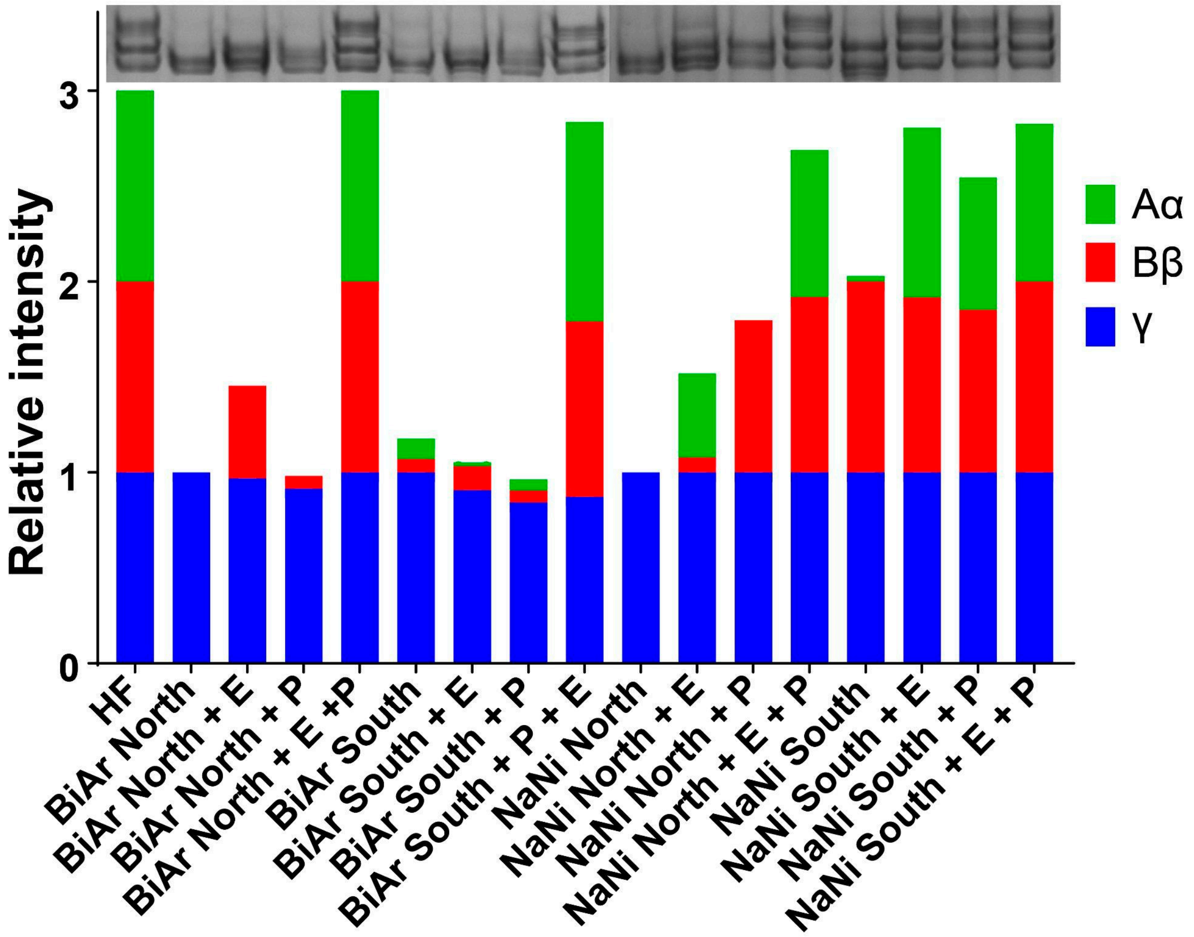

Figure S2: Fibrinogenolytic activity of *B. arietans* and *N. nigricollis* venoms from northern and southern Nigerian snake samples. Here, HF: human fibrinogen control, BiAr: *B. arietans,* NaNi: *N. nigricollis,* E: EDTA, P: PMSF.

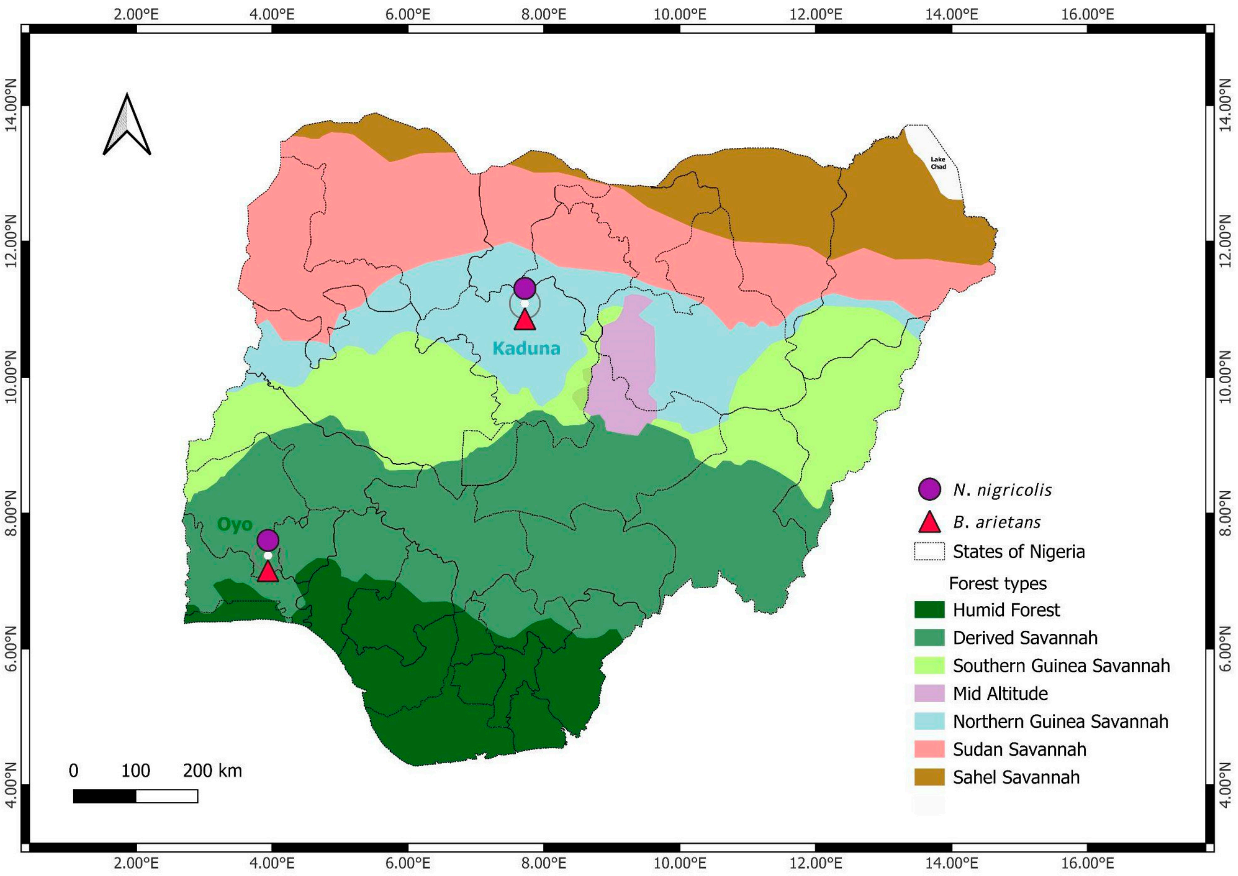

Figure S3: Map of Nigeria depicting various forest types and locations of snake venom collection. Purple circles indicate *N. nigricolis*, while red triangles represent *B. arietans*. Forest type data were obtained from the Africa Soil Information System (AfSIS) at https://africasis.isric.org and visualized using QGIS 3.40.
