# Supplementary material for: Preliminary Insights into Geographic Variation in Venom Profiles and Functional Activities of Nigerian Snakes, Bitis arietans and Naja nigricollis
Source: Toxins (Basel). 2026 May 7;18(5):221. doi: 10.3390/toxins18050221 (PMC13211584; doi:10.3390/toxins18050221)
Supplement: Supplementary file 1 [file toxins-18-00221-s001.zip › toxins-4261761_supplementary_table.pdf]

Table S1. Concordance between venom proteomes across northern and southern venom samples of Nigerian snakes (*B. arietans* and *N. nigricollis*).

Species	Spearman			Jaccard		
	rho_ci	p_asym	p_perm_fmt	Intersection	Union	Jaccard coefficient
B. arietans	0.466 (95% CI -0.133–0.880)	4.4e-02	0.0451	14	19	0.74
N. nigricollis	0.918 (95% CI 0.706–0.998)	7.6e-08	< 1e-4	16	18	0.89

In this table, Spearman's rank correlation (ρ) tests the association between family-level abundances in the northern and southern venoms for each species; 95% CIs are shown in parentheses. P_asym is the two-sided asymptotic p-value for the null hypothesis $\rho = 0$; p_perm is the permutation p-value. Jaccard coefficient quantifies presence/absence agreement between the northern and southern venoms as Intersection ÷ Union, where "Intersection" is the number of toxin families detected in both datasets and "Union" is the total unique families detected across either dataset. Higher ρ and Jaccard indicate stronger quantitative and qualitative concordance, respectively.

Table S2. Family-level differences in toxins between northern and southern venom samples of Nigerian snakes (*B. arietans* and *N. nigricollis*).

Species	Family	North	South	Effect size
B. arietans	Disintegrin	4	0	-4
	AP	2	5	3
	CTL	16	13	-3
	SVSP	16	19	3
	VCC3	0	3	3
	Cathepsins	2	0	-2
	Kunitz	1	3	2
	LAAO	3	5	2
	BPP	0	1	1
	Cystatins	1	2	1
	Serpin	0	1	1
	SVMP (PIII)	9	8	-1
	5'-NTD	2	2	0
	CRVP	1	1	0
	PDE	2	2	0
	PLA_2	1	1	0
	PLB	1	1	0
	SVMP (PII)	1	1	0
	VEGF	1	1	0
N. nigricollis	C-3FTx	9	7	-2
	N-3FTx	5	3	-2
	AP	1	0	-1
	CRVP	2	3	1
	Serpin	0	1	1
	SVMP (PIII)	8	7	-1
	5'-NTD	1	1	0

	CTL	1	1	0
	GP	1	1	0
	Kunitz	2	2	0
	LAAO	4	4	0
	NGF	1	1	0
	PDE	1	1	0
	PLA_2	11	11	0
	PLB	1	1	0
	SVMP (PII)	1	1	0
	SVSP	1	1	0
	VCC3	1	1	0

In this table, for each species, the abundances of each toxin family in the northern and southern venoms were compared to quantify venom proteome discrepancy. “Effect size” represents the signed difference (South – North), where positive values (right of 0) show higher abundance in the southern region; negative values (left of 0) show higher abundance in the northern region. Here, VCC3: venom complement C3; SVSP: snake venom serine proteinase; LAAO: L-amino-acid oxidase; kunitz: venom kunitz-type family; BPP: bradykinin-potentiating peptide; AP: aminopeptidase; CRVP: Cysteine-rich venom protein; VEGF: venom endothelial growth factor; SVMP: snake venom metalloproteinase; PLB: phospholipase B; PLA_2: phospholipase A2; PDE: phosphodiesterase; 5′-NTD: 5′-nucleotidase; CTL: c-type lectin; GP: glutathione peroxidase; C-3FTx: cytotoxic 3-finger toxin; N-3FTx: neurotoxin 3-finger toxin.
